# Sex- and genotype-dependent nicotine plus cue-primed reinstatement is enhanced in adolescent Sprague Dawley rats containing the human *CHRNA*6 3′-UTR polymorphism (rs2304297)

**DOI:** 10.3389/fpsyt.2022.1064211

**Published:** 2023-01-10

**Authors:** Diana Carreño, Shahrdad Lotfipour

**Affiliations:** ^1^Department of Pharmaceutical Sciences, University of California, Irvine, Irvine, CA, United States; ^2^Department of Pathology and Laboratory Medicine, University of California, Irvine, Irvine, CA, United States; ^3^Department of Emergency Medicine, University of California, Irvine, Irvine, CA, United States

**Keywords:** alpha6 nicotinic acetylcholine receptor subunits, addiction, drug-seeking behavior, pharmacogenetics, relapse

## Abstract

**Rationale:**

Large-scale human candidate gene studies have indicated that a genetic variant (rs2304297) in the alpha(α)6 nicotinic acetylcholine receptor (nAChR) subunit, encoded by the *CHRNA*6 gene, may play a key role in adolescent nicotine addictive behavior. We hypothesized that the polymorphism selectively enhances nicotine + cue-primed reinstatement, but not nicotine- or cue-reinstatement in α6*^GG^* (risk) vs. α6*^CC^* (non-risk) allele carriers, without having baseline effects on natural rewards.

**Methods:**

Using CRISPR-Cas9 genomic engineering, we developed a humanized rat line with the human gene variant of the *CHRNA*6 3′-UTR*^C^*^123^*^G^* polymorphism in Sprague-Dawley rats. Genetically modified adolescent male and female rats were food trained under a fixed-ratio (FR)1 schedule of reinforcement and progressively increased to FR5. Animals were implanted with catheters and began nicotine self-administration (15 μg/kg/infusion) at FR5. Upon reaching stable responding, reinforced behavior was extinguished by removal of drug and cues. Reinstatement testing began for cue only, nicotine only, and nicotine + cue in a Latin Square Design. Animals were returned to extinction conditions for 2 days minimum between testing.

**Results:**

For natural food rewards, nicotine self-administration, progressive ratio, and extinction, adolescent male and female (α6*^GG^* and α6*^CC^*) rats exhibited equivalent behaviors. Male α6*^GG^* rats show enhanced nicotine + cue-primed reinstatement when compared with male α6*^CC^* rats. This genotype effect on reinstatement was not seen in female rats.

**Conclusion:**

Our findings support the *in vivo* functional role of the human *CHRNA*6 3′-UTR SNP genetic variant in sex-dependently enhancing nicotine seeking behavior in adolescent rats. Overall, the findings support clinical and preclinical data highlighting a role of α6 nAChRs mediating sex heterogeneity in substance use and related phenotypes.

## 1. Introduction

In recent years the prevalence of adolescent electronic nicotine use has dramatically increased (1, 2). Adolescent electronic nicotine use remains a public health concern given that its use can progress into combustible cigarette smoking and conditions the developing brain for addiction to other drugs of abuse (3–6). Young people are highly sensitive to nicotine, exhibiting symptoms of dependence soon after smoking initiation and before the start of daily smoking (7). Further, electronic cigarette cessation interest has increased among adolescents. In 2019 an estimated 57.5% middle and high school (about 3.5 million) students using tobacco/nicotine made an attempt to quit (8). The prevalence of unsuccessful quit attempts among middle and high school students who had either used e-cigarettes or cigarettes, was higher in 2020 than previous years (9).

Nicotine is the principal reinforcing constituent in tobacco products responsible for drug seeking and addiction. Nicotine directly activates neuronal nicotinic acetylcholine receptors (nAChR), which are ligand-gated ion channels consisting of five membrane-spanning subunits located on several neurotransmitter systems in the brain, most notably dopaminergic neurons (10). In the mammalian brain the predominant and most prevalent nAChRs implicated in the addictive properties of nicotine are those containing α4 and β2 subunits (11–13). These subunits can independently partner with α6* nAChR subunits to makeup α6β2β3 (non-α4) and α6α4β2β3 nAChRs (10, 13–17). α6* nAChRs are largely localized in expression on the cell body or terminal regions of dopaminergic neurons and act as critical modulators of dopamine release in reward regions of the brain (10, 14, 17, 18). α6* nAChRs reach highest mRNA expression in the ventral tegmental area (VTA) and substantia nigra (SN) during adolescence (19). α6 knockout (KO) mice do not self-administer nicotine even with an extensive range of doses, an effect which can be rescued using lentiviral re-expression of α6 nAChR subunits directly within the VTA (20). Further, administration of alpha(α)-conotoxin MII, an α6β2β3 antagonist, into the nucleus accumbens shell attenuate nicotine self-administration in rats (21). These studies suggest the α6* nAChRs in the VTA and/or its projections, e.g., nucleus accumbens and prefrontal cortex, are necessary and sufficient to establish nicotine self-administration. Evidence also demonstrates that a selective α6 nAChRs antagonist, bPiDDB, is able to decrease nicotine-induced reinstatement in nicotine self-administering rats (22). Drug paired cues are likely important factors in such behavioral effects, as intra-VTA infusion of an α6 nAChRs antagonist, α-conotoxin MII, blocks the rewarding effects of cues paired with a drug reinforcer (23, 24). Such studies are important as cue and drug-induced reinstatement is a preclinical model of drug-seeking behavior (see reviews (25–27)) and highlight the importance of α6* nAChRs critical involvement.

Clinically, a large body of literature highlights that a single nucleotide polymorphism (SNP) in the α6 nAChR subunit (encoded by the *CHRNA*6 gene) is associated with nicotine/tobacco use and related problems (28–39). The human *CHRNA*6 SNP is located in the 3′-untranslated region (UTR), a genomic region known to regulate mRNA stability, localization, and translation (40, 41). The α6 nAChR subunit SNP has been associated with increased cigarette smoking and drug experimentation during adolescence with α6*^GG^*- more impacted than α6*^CC^*-allele carriers (34). Additionally, a clinical study revealed a sex-treatment interaction for rs230497, with a two-fold greater abstinence in the bupropion arm in males versus females by end of treatment, although not surpassing Bonferroni corrections (33). Sex heterogeneity has been observed as a critical factor to be considered with α6 nAChRs, the *CHRNA*6 gene and the *CHRNA*6 3′-UTR SNP in nicotine addiction and other neurological diseases (33, 42–45). The *CHRNA*6 3′-UTR SNP has been associated with nicotine dependence in males (46). Further, other *CHRNA*6 SNPs in general show greater nicotine dependence associations in males than females (43). As such, clinical findings provide evidence for the *CHRNA*6 3′-UTR SNP contributing to the susceptibility of tobacco/nicotine dependence with the strong likelihood of sex-dependent effects. Our prior pre-clinical data support this hypothesis (47).

Mechanisms underlying how the *CHRNA*6 3′-UTR mediates adolescent substance use are not known. To investigate the human *CHRNA*6 3′-UTR SNP *in vivo*, our lab generated a novel, humanized rodent line. Our lab replaced the entire *CHRNA*6 3′-UTR of the rat line with the human *CHRNA*6 3′-UTR, generating a translational model of α6 nAChRs (47). Our recent results using this novel rat line confirm *in vivo* functionality of the *CHRNA*6 3′-UTR SNP, with genotype- and sex-dependent effects observed on adolescent nicotine-induced behaviors (47). The aim of our current study is to assess sex- and genotype-dependent effects on the influence of nicotine self-administration, progressive ratio, extinction and reinstatement of nicotine-seeking behavior in adolescent males and females containing the humanized *CHRNA*6 3′-UTR SNP.

## 2. Materials and methods

### 2.1. Generation of human CHRNA6 3′-UTR SNP rodents

Human *CHRNA*6 3′-UTR SNP knock-in rats were designed and created with CRISPR/Cas9 gene editing techniques by Cyagen Biosciences as described in Cardenas et al. (47). Briefly, donor vectors and sgRNA were designed to target the 3′-UTR of the rat *CHRNA6* gene (GenBank accession number: NM_057184.1; Ensembl: ENSRNOG00000012283). Donor vectors contained the human *CHRNA*6 3′-UTR (559 nucleotides) with either the minor SNP rs2304297 allele, C, or major SNP rs2304297 allele, G, at nucleotide position 123 and replaced the rat 3′-UTR (95 nucleotides).

### 2.2. Animals

**FIGURE 1:**
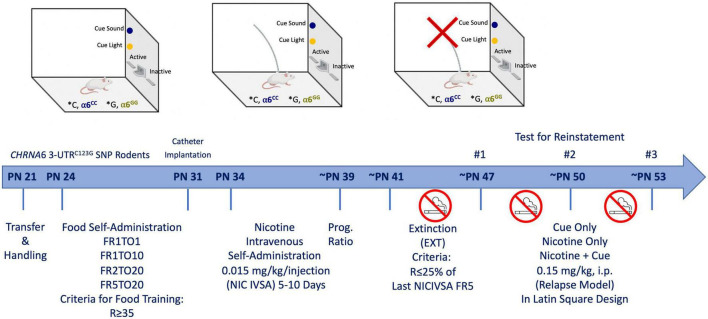
Experimental paradigm. Male and female *CHRNA6* 3′-UTR SNP knock-in rats underwent food self-administration, catheter implantation, nicotine self-administration, progressive ratio, extinction, and cue-, nicotine-, and nicotine + cue-induced reinstatement.

Male and female wild type (WT) Sprague-Dawley rats were purchased from Charles river and bred in house with human *CHRNA*6 3′-UTR*^C123G^* SNP rats. Upon weaning at postnatal day (PN) 21, animals were transferred out of the mother’s cage and separated by sex (Figure 1). All animals were handled for 3-days prior to experimentation and group-housed (2 per cage) throughout the experiment. All rats were maintained on a 12-h light/dark cycle (lights on at 07:00 am). All experimental procedures were in compliance with NIH guidelines and were approved by the Institutional Animal Care and Use Committee of the University of California, Irvine. Animals were handled for 2 min daily before testing began. No more than one male and female animal per litter per experimental group was used to avoid potential confounds. Rats were minimally food-restricted beginning two days prior to operant conditioning to increase the motivation to learn the self-administration paradigm similar to our prior study (48). Group-housed (2 per cage) adolescent rats were fed 15–25 g of food to maintain normal growth during self-administration testing (49). Animal weights based on postnatal days are presented in Supplementary Figure 1, with postnatal days across condition highlighted in Supplementary Figure 2. Attrition rates for animals in our current study are shown in Table 1. Similar to our previous wild type Sprague Dawley nicotine reinstatement studies in adolescent rats (48) we did not administer vaginal swabs in the present study to assess estrous cycle (50–52). The age range of adolescence was defined based on the prior studies of Spear (53).

**TABLE 1:** Attrition rates for *CHRNA*6 3′-UTR SNP rats in the study.

Condition	α6^CC^ F	α6^GG^ F	α6^CC^ M	α6^GG^ M
Food reinforcement			1	2
Surgery complication	5	4	2	4
NIC IVSA	10	8	14	9
Reinstatement outliers	2	1	2	3
Reinstatement testing (Final ‘N’)	10	14	11	8

### 2.3. Apparatus

Animals were tested in plexiglass operant chambers (Med Associates, St Albans, VT, USA), equipped with two levers. The required number of responses at the reinforced (Reinf) lever turned on a cue light over the lever, turned off the house light, and activated an externally mounted syringe pump that infused drug. During the infusion (5.6 s yielding 100 μl of solution) and timeout period (20 s) the cue light remained illuminated, and the house light remained off.

After the timeout period, the house light turned on and signaled the availability of a reinforcer. Responses on the non-reinforced (Non-Reinf) lever were recorded but had no consequences. Procedures modeled previous work (54, 55).

### 2.4. Food self-administration

To facilitate learning, male and female adolescent (PN 24) rats were trained twice per day in a 30 min session to lever press for food pellets (45 mg rodent purified diet; Bio-Serv, Frenchtown, NJ, USA) in lever pressing operant testing chambers (Med Associates, St. Albans, VT, USA), based on previous studies (54, 56) (Figure 1). One wall of the chamber contained two levers, a cue light over each, and a house light. The right lever was assigned as the active (Reinf) lever, each response at which was rewarded with delivery of food and presentation of cue light and tone. The left lever was inactive (Non-Reinf) had no consequences but was recorded as a measure of non-specific activity. The animals started at an FR1TO1 (fixed-ratio 1, 1s timeout) schedule of reinforcement, followed by FR1TO10, FR2TO20 and finally FR5TO20, progressing upon earning 35 reinforcers.

### 2.5. Surgery

Following successful acquisition of food training, rats were anesthetized with Equithesin (0.0035 ml/g body weight) and implanted with indwelling jugular vein catheters (57–59) (Figure 1). After surgery, rats were given the analgesic carprofen (5 mg/kg, subcutaneous). During the 2–3-day recovery period, catheters were flushed daily with heparinized saline solution (1 ml of 1,000 units/ml heparin into 30 ml of bacteriostatic saline) to maintain patency. Catheter patency was tested for rapid (5–10 s) anesthesia by infusing propofol (5 mg/kg, i.v.) before and after the completion of self-administration experiments. Only animals showing rapid anesthesia were included in analyses.

### 2.6. Nicotine intravenous self-administration and extinction

Animals (PN 34) intravenous self-administered (IVSA) nicotine (0.015 mg/kg/infusion) at an FR5 schedule for 1-h daily session for a minimum of 5 days, or until they reached stable responding (Reinforced responses (Reinf) within 20% of the mean over the last 3 days; R ≥ 2 × Non-Reinforced responses; R ≥ 5) (48) (Figure 1). A compound stimulus, light and tone, were paired upon delivery of nicotine infusion. A dose of 0.015 mg/kg/infusion was chosen based on previous adult and adolescent studies (54, 55). Baseline responding was defined as the average reinforced responses over the last three days of self-administration. Rats were then allowed to respond to at the dose of 0.015 mg/kg/infusion on Progressive Ratio (PR) schedule (∼PN 39) (Figure 1). The PR schedule of reinforcement is a measure of motivation to obtain the drug (60). The sequence was determined using the exponential formula (5 exp (0.2 × infusion number)-5) such that the required responses per infusion were as followed: 1, 2, 4, 6, 9, 12, 15, 20, 25, 32, 40, 50, 62, 77, 95, 118, 145, 178, 219, 268, 328, 402, 492 (60). PR conditions were the same for FR sessions, with the exception that the sessions were 4-h duration. Breakpoint was achieved when >20 min of inactivity on the active lever elapsed. After reaching stable responding and two-day of PR schedule extinction-reinstatement testing began.

During extinction (∼PN 41), animals were placed in the same operant testing chambers the animals were not connected to the infusion tubing, the house light remained on and responses on the levers were counted but had no consequences, as such no cues or reward were delivered (Figure 1). Extinction sessions were 1-h per day for a minimum of 5 days, or until responding was reduced to 25% of baseline (48).

### 2.7. Cue and nicotine-induced reinstatement

After meeting extinction criteria, reinstatement testing began (∼PN 47) (48) (Figure 1). Nicotine-seeking was reinstated using three reinstatement conditions given in a within-subjects counterbalanced design: Cues, nicotine-primed alone, and nicotine-primed paired with cues. Presentation of cues consisted of cue light illumination and sound in the testing chamber. Nicotine-prime injections contained 0.15 mg/kg nicotine and was administered intraperitoneally immediately before the reinstatement test. The nicotine-prime dose was chosen based on previous work (26, 54). Between reinstatement tests, animals were returned to extinction condition for a minimum of two days, or until extinction criteria were met. Reinstatement was defined as a significant increase in responding from the last day of extinction.

### 2.8. Data analysis

Data were analyzed using JMP (SAS Institute) software (48). Food acquisition was analyzed by a compound 4-way multivariate ANOVA for lever presses (Reinf and Non-Reinf) × sex (male and female) × Genotype (α6*^GG^* and α6*^CC^*) × FR schedule (FR1TO1, FR1TO10, FR2TO20, and FR5TO20) with repeated measures on lever presses and FR schedule, with Bonferroni corrected t-test *post hoc* comparisons. Nicotine self-administration data were analyzed by a compound 4-way multivariate ANOVA Reinf/Non-reinf responses × day × sex (male and female) genotype (α6*^GG^* and α6*^CC^*) × day (day 3–5) with repeated measures on Reinf/Non-reinf responses and day. Reinstatement data were analyzed as normalized reinforced responding. Mean responses for reinstatement condition were analyzed by a 3-way multivariate ANOVA for sex x genotype (α6*^GG^* and α6*^CC^*) × reinstatement condition (cue only, nicotine only, and nicotine plus cues), with repeated measure on reinstatement condition. Significant main effects were further analyzed with 1-way ANOVAs and Bonferroni-corrected paired or unpaired t-tests, as appropriate. Food reinstatement data were analyzed as normalized reinforced responding. Mean responses for reinstatement condition were analyzed by a 3-way multivariate ANOVA for sex x genotype (α6*^GG^* and α6*^CC^*) × reinstatement condition (cue only, food only, and food plus cues), with a repeated measure on reinstatement condition. Significant main effects were further analyzed with 1-way ANOVAs and Bonferroni-corrected paired or unpaired *t*-tests, as appropriate.

## 3. Results

### 3.1. The *CHRNA6* 3′-UTR SNP knock-in does not impact food self- administration in male and female rats

**FIGURE 2:**
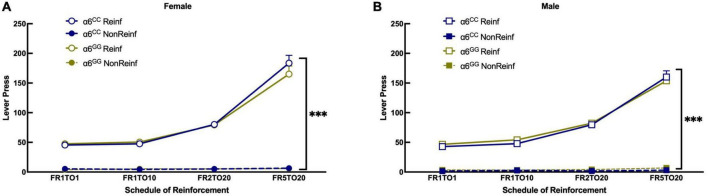
The human *CHRNA*6 3′-UTR SNP knock-in does not impact food self-administration. Female **(A)** and male **(B)**, α6^CC^ and α6^GG^, mean daily 30 min responses ± SEM for food self-administration at Fixed Ratio (FR)1 Time out (TO)1, FR2TO10, FR2TO20, FR5TO20 schedules of reinforcement. ***p = 0.0001 Reinforced (Rein) vs. Non-Reinforced (Non-reinf) responses. *N* = 8–14/group. Circles represent females and squares represents males.

To facilitate acquisition of lever pressing, adolescent male and female α6^GG^ and α6^CC^ rats were evaluated for food reinforcement at Fixed Ratio (FR)1 timeout (TO)1 and then escalated to higher schedules of reinforcement at FR1TO10, FR2TO20, and FR5TO20. A 4-way ANOVA revealed a significance for Reinf/Non-Reinf lever presses [*F*(1,39) = 1169.4560, *p* < 0.0001] and FR schedule [*F*(3,117) = 295.4014, *p* < 0.0001]. A significant interaction was observed between Reinf/Non-Reinf lever presses x FR schedule [*F*(1,117) = 302.9465, *p* < 0.0001]. Data are collapsed by sex since there was no sex or genotype interaction, but graphs (Figures 2A, B) are shown separately by sex and genotype for clarity. Male and female adolescent α6^GG^ and α6^CC^ equally learn to press a lever for a natural reward.

### 3.2. No sex or genotype differences for intravenous self-administration, progressive ratio, and extinction in male and female adolescent *CHRNA6* 3′-UTR SNP knock-in rats

**FIGURE 3:**
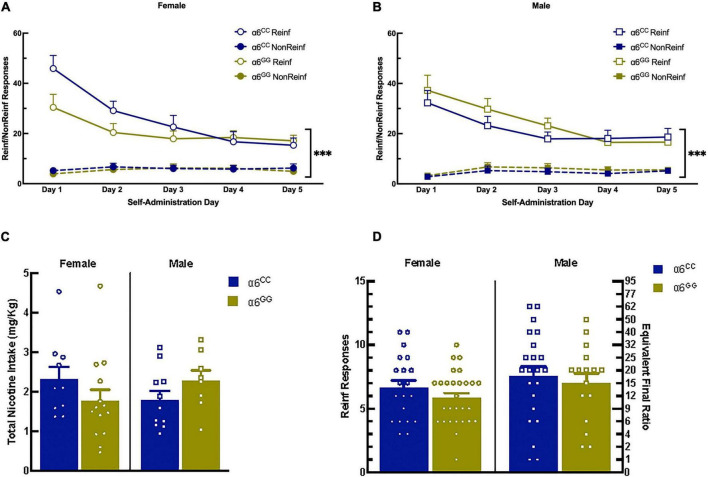
The human *CHRNA*6 3′-UTR SNP knock-in does not impact nicotine self-administration, progressive ratio, and nicotine intake. Female **(A)** and male **(B)**, α6^CC^ and α6^GG^, mean daily 1-h responses ± SEM for nicotine self-administration at FR5TO20 schedule of reinforcement. No genotype or sex differences were observed for nicotine intake **(C)** or breakpoint values **(D)**. ***p < 0.001. *N* = 8–14/group. Circles represent females and squares represent males.

**FIGURE 4:**
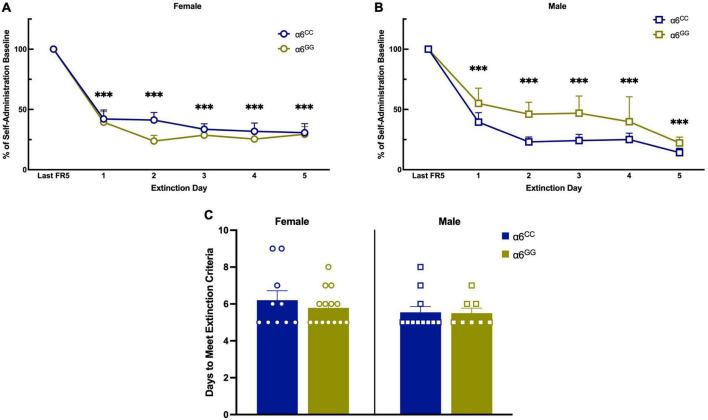
The human *CHRNA*6 3′-UTR SNP does not influence extinction. After completion of nicotine self-administration, female **(A)** and male **(B)**, α6^CC^ and α6^GG^, were allowed to respond on the reinforced and non-reinforced lever without schedule consequence (e.g., infusion of nicotine, cue light and tone). Data are presented as a mean ± SEM percent of the last day of nicotine self-administration responding. No genotype or sex differences were observed for days to meet extinction criteria **(C)**. ***p < 0.001 vs. Last FR5. *N* = 8–14/group. Circles represent females and squares represent males.

During nicotine intravenous self-administration there was a significant effect of Reinf/Non-reinf responses for days 1–5 [(*F*1,39) = 183.4430, *p* = 0.0001)], Day [*F*(4,36) = 11.7757, *p* = 0.0001], interaction between Reinf/Non-reinf responses and Day [*F*(4,36) = 19.2790, *p* = 0.0001] (Figures 3A, B). No sex or genotype effects were observed for total nicotine intake (Figure 3C) or breakpoint values (Figure 3D). Following stable nicotine self-administration, nicotine-seeking behavior was extinguished by removal of nicotine and associated cues. All animals significantly reduced their responding on the reinforced lever beginning on Day 1 and continued throughout extinction (Figures 4A, B). No sex differences were observed for days to meet extinction criteria as shown in Figure 4C, males and females showing equivalent 25% or on less last day of extinction responding with an average of 6 days.

### 3.3. Sex- and genotype-dependent effects on reinstatement of nicotine in the *CHRNA6* 3′-UTR SNP adolescent rats

**FIGURE 5:**
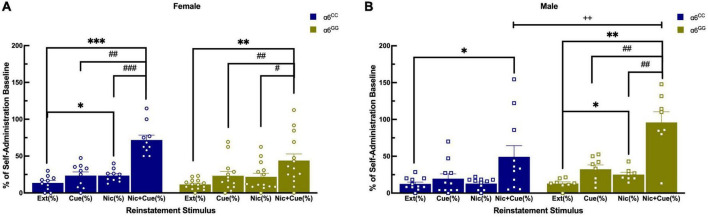
Sex and genotype dependent effects of reinstatement of nicotine in humanized *CHRNA*6 3′-UTR SNP rats. α6 3′ UTR SNP genotype- and sex-dependently influences nicotine + cue primed reinstatement, with males more impacted than females based on genotype **(A,B)**. ***p < 0.001, **p < 0.01, *p < 0.05 vs. Extinction; ^#^*p* < 0.05, ^##^*p* < 0.01, ^###^*p* < 0.001 vs. Nicotine + Cue; ^+ +^*p* = 0.01 Male α6^GG^ compared with α6^CC^. N = 8–14/group. Circles represent females and squares represent males.

Following extinction, animals were triggered to reinstate to nicotine-seeking behavior with cues-, nicotine- and combination of nicotine plus cues (Figures 5A, B). For reinstatement overall ANOVA reveal a sex x genotype interaction [*F*(1,38) = 8.0052, *p* = 0.0074], data was separated by sex and genotype. Main effects of reinstatement stimuli were found [*F*(3,36) = 26.0462, *p* < 0.0001] and a trend for reinstatement stimuli x sex x genotype [(*F*(3,36) = 2.6865, *p* = 0.061]. Further one-way ANOVA for nicotine + cue reveal [*F*(1,39) = 10.0598, *p* = 0.0030] and a one-tail t-test reveal male α6^GG^ show enhanced nicotine + cue primed reinstatement as compared to α6^CC^ (*p* = 0.01) (Figure 5B). In females, α6^CC^ rats show a trend for enhanced nicotine + cue primed reinstatement as compared with α6^GG^ rats (*p* = 0.08) (Figure 5A).

## 4. Discussion

We have previously demonstrated equivalent adolescent WT Sprague Dawley male and female behavior in the acquisition of a natural and drug reward and nicotine seeking behavior using a reinstatement model (48). In the current study, we investigate the role of humanized *CHRNA*6 3′-UTR SNP knock-in in male and female adolescent rats. Our results show no sex or genotype effects for food self-administration, nicotine self-administration, progressive ratio, and extinction. We observe a sex and genotype bidirectional effect during reinstatement testing specifically, α6^GG^ males exhibit enhanced nicotine + cues reinstatement when compared to α6^CC^ males and a trending for α6^CC^ when compared to α6^GG^ females. Our study is the first to use an *in vivo* translational model of nicotine behavioral phenotype during adolescence. The current study uses an age range for male and female rats between PN 28–42 as the typical period of adolescence (53). Ages in the “gray zone,” i.e., earlier than PN 28 and later PN 42 have been considered as part of adolescence up to around PN 60 (53). Thus, our studies were done during adolescence, a gradual transition period of soft events with sexual maturity as part of a developmental milestone (53).

### 4.1. The *CHRNA6* 3′-UTR SNP knock-in does not influence a natural reward

The *CHRNA*6 3′-UTR SNP knock-in does not influence the ability for male and female, α6^GG^ and α6^CC^, adolescents to acquire and maintain food self-administration. Our results are in accord with our recent manuscript using a separate cohort characterizing the *CHRNA*6 3′-UTR SNP knock-in rats (47) and in adolescent wild type Sprague Dawley rats (48). These studies show that age matched male and female wild type and *CHRNA*6 3′-UTR SNP knock-in rats do not exhibit sex effects. Comparing food reinforcement between *CHRNA*6 3′-UTR SNP and wild type rats show similar behavior at different schedules of reinforcement, with the exception of slightly higher FR5TO20 for the 3′-UTR SNP rats (48). Assessing palatable food conditioned place preference (CPP) in α6 KO and WT C57BL/6J(B6) mice, exhibited similar place preference scores for the context associated with palatable food suggesting the α6 inactivation does not result in the incentive value of a natural reward (61). Intra-VTA perfusion of α-CntxMII did not yield an effect on food self-administration in adult male rats on a fixed-ratio schedule of reinforcement (15). Additionally, administration of a range of doses for r-bPiDI (2.47-74 μmol/kg; s.c.), a selective small molecule antagonist of α6β2* nAChR, a tertiary amino analog, 1,10-bis(3-methyl-5-6,-dihydropyridin-1(2H)decane (r-bPiDI), derived from N,N-decane-1,10-diyl-bis-3-picolinium diiodide (bPiDI), did not alter the number of food pellets earned in male rats (62). Future studies should examine the role of a6* nAChR subunit in food-seeking behavior given the mechanism for food self-administration may differ from food-induced reinstatement.

### 4.2. The *CHRNA6* 3′-UTR SNP knock-in does not influence nicotine intravenous self-administration, progressive ratio, and extinction

In our current studies, the *CHRNA*6 3′-UTR SNP knock-in does not influence nicotine self-administration in adolescent (PN 34) males and females α6^GG^ and α6^CC^ at a dose of 0.015 mg/kg/injection on a fixed ratio and progressive ratio schedule of reinforcement. Our results are consistent with adolescent male and female Sprague Dawley WT rats (PN 34), which exhibit similar nicotine self-administration in an equivalent paradigm and show lack of sex effect (48). Comparing *CHRNA*6 3-UTR SNP to age matched wild type rats show higher nicotine self-administration and nicotine intake (48). This is likely influenced by the higher responses at FR5TO20 for food reinforcement prior to nicotine self-administration, as progressive ratio values were similar between *CHRNA*6 3′-UTR SNP and age matched wild type rats (48). Decreased nicotine self-administration in both, fixed ratio and progressive ratio have been observed with microinjections into either the nucleus accumbens shell or the ventral tegmental area with an α6β2* nAChR antagonist in rats (15, 21). Intra-VTA α-CntxMII pretreatment significantly reduced responding for nicotine self-administration at a dose of 0.03 mg/kg/infusion in adult male WT Sprague Dawley rats suggesting the role of α6* nAChR in nicotine-reinforcing properties (15). Further, in adult rats, nicotine self-administration was decreased with a selective antagonist of α6β2* nAChR, r-bPiDI, at a dose of 0.03 mg/kg/infusion (62). Additionally, bPiDI prevented the acquisition of nicotine self-administration in WT and α4-S248F mice, mutant α4* nAChR that are insensitive to blockade by mecamylamine, a non-selective nicotinic receptor antagonist (63). These studies suggest the involvement in α6* nAChR in nicotine reinforcement, particularly at higher nicotine doses that were not assessed in our current studies. Future studies are needed to examine dose-, sex-, or genotype-dependent effects, along with α6* nAChR blockade to suppress nicotine self-administration in the adolescent humanized *CHRNA*6 3′-UTR SNP rats. Whereas our results suggest that adolescent male and female α6^GG^ and α6^CC^ have equivalent nicotine self-administration and reward response at the 0.015 mg/kg/injection, a dose response is warranted. It is possible that nicotine pharmacodynamics and pharmacokinetic properties may be dose-dependently shifted in male and female humanized *CHRNA*6 3′-UTR SNP rats, which could be assessed in future studies.

Our results illustrate that male and female adolescent containing the *CHRNA*6 3′-UTR SNP exhibit equivalent extinction learning. These effects are similar to age matched wild type rats (48). Both male and females, α6^GG^ and α6^CC^, exhibited extinguished lever-pressing behavior beginning at day 1 and decreasing over time. Self-administration, extinction/reinstatement paradigm in adolescent males (PN 45) and young adult (PN 58) provides an understanding of the molecular mechanisms of extinction learning revealing key structures involved including the mPFC, OFC, Nucleus accumbens (NAc, core and shell) and amygdala (64). Further research is needed to understand how dose-dependent nicotine levels alter learning and memory processing during extinction in the humanized male and female *CHRNA*6 3′-UTR SNP rats.

### 4.3. Sex- and genotype dependent effects with a greater impact in α6^GG^ males versus α6^CC^ males

For nicotine-induced reinstatement, male α6^GG^ and female α6^CC^ rats illustrated enhanced nicotine combined with cue responding with males being more impacted. Cue only failed to reinstate responding in all groups. In age matched wild type rats, cue-induced reinstatement was observed in both males and females (48). Male α6^GG^ and female α6^CC^ rats show that a non-contingent administration of nicotine during extinction of nicotine self-administration reinstates responding. These results are in accord with our recent studies using adolescent male and female wild type Sprague Dawley rats (48). There is mounting evidence for significant strain-dependent differences for nicotine self-administration (65, 66). Whereas we observe no sex or genotype differences for natural-, drug-reward and extinction between wild type Sprague Dawley and the *CHRNA6* 3′-UTR SNP rats with a Sprague Dawley genetic background, our reinstatement results suggest otherwise. In particular, male α6^GG^ rats show the greatest nicotine + cue-induced reinstatement as compared to age matched wild type rats (48). The strain difference between the WT Sprague Dawley and the *CHRNA6* 3′-UTR SNP is the replacement of the rat *CHRNA6* 3′-UTR with the human *CHRNA6* 3′-UTR. Polymorphisms in 3′-UTR are known to be associated with neurological disorders and behaviors via involvement of micro(mi)RNAs and RNA binding protein (RBP) by influencing mRNA translation (67, 68). The human *CHRNA6* 3′-UTR may bind to miRNA present in humans, but not rodents. The technology to understand the mechanism of the *CHRNA6* 3′-UTR across species in mediating nicotine-induced behavioral sensitization is limited (69).

Assessing α6* nAChR mRNA and protein expression could shed light into lack of sex or genotype behavioral differences. Sub-chronically treated humanized *CHRNA*6 3′-UTR SNP rats, following a 4-day nicotine pretreatment paradigm, found no sex-, genotype- or nicotine-dependent alterations in α6 nAChR mRNA expression; however, behavior test showed α6^CC^ females and α6^GG^ males exhibited nicotine induced locomotor and anxiolytic behavior compared to their saline-treated counterparts (47). Further, in adult male and female mice with fluorescently tagged α4 and α6 nAChRs showed no sex differences for CPP scores at 0.5 mg/kg nicotine s.c. however, there was a significant correlation between upregulation of α4α6, but not α6 (non-α4) nAChRs and reward related behavior in males versus females (70). Future studies are essential in assessing neuronal activation, α6* nAChR mRNA and protein expression following nicotine + cue reinstatement in adolescent *CHRNA*6 3′-UTR SNP male and female rats.

## 5. Conclusion

Taken together, our data suggest no genotype and/or sex effects were observed for natural food reinforcement, five-day total drug intake for nicotine self-administration, progressive ratio schedules of reinforcement, extinction behavior, or other parameters of reinstatement (i.e., cue-primed). Our results suggest that adolescent male α6^GG^ (and potentially female α6^CC^) rats may be at risk for addictive effects as assessed by reinstatement paradigm. Future studies will need to evaluate the role of nicotine dose, age, and sex-dependent effects during adulthood. Further assessments of the neurobiological mechanisms involved are also warranted in order to identify what drives the nicotine-induced effects in our *CHRNA*6 3′-UTR SNP line in a sex- and genotype dependent manner. Such work may assist in translational studies in humans for improved prevention and intervention strategies to curb nicotine-seeking behavior.

## Data availability statement

The original contributions presented in this study are included in the article/Supplementary material, further inquiries can be directed to the corresponding author.

## Ethics statement

This animal study was reviewed and approved by Institutional Animal Care and Use Committee of the University of California, Irvine.

## Author contributions

DC collected data and conducted the literature review. Both authors conceived the study design, conducted data analysis, contributed to the manuscript writing, contributed to the critical revision of data analysis, and approved the final version of the manuscript.

## Supplementary material

Supplementary Figure 1 Body weight (g) across postnatal days. Male and female α6^GG^ and α6^CC^. Circles represent females and squares represent males.

Supplementary Figure 2 Postnatal day across nicotine self-administration, extinction, and reinstatement. No sex differences for the age of initiation of behavior for nicotine self-administration, progressive ratio, extinction, and reinstatement. Mean ± SEM postnatal days across study conditions for male and female α6^GG^ and α6^CC^. Circles represent females and squares represents males.
