# Supplementary figures and images for: Sex- and genotype-dependent nicotine plus cue-primed reinstatement is enhanced in adolescent Sprague Dawley rats containing the human CHRNA6 3′-UTR polymorphism (rs2304297)
Source: Front Psychiatry. 2023 Jan 10;13:1064211. doi: 10.3389/fpsyt.2022.1064211 (PMC9872558; doi:10.3389/fpsyt.2022.1064211)

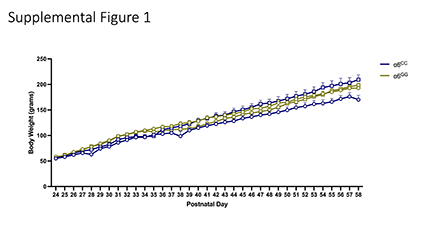

Supplement: Supplementary Figure 1 — Body weight (g) across postnatal days. Male and female α6GG and α6CC. Circles represent females and squares represent males. [file Image_1.TIFF]

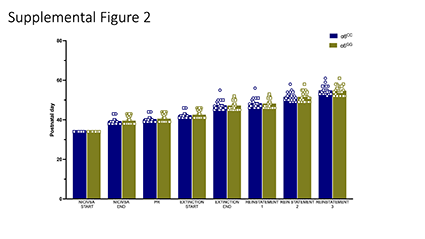

Supplement: Supplementary Figure 2 — Postnatal day across nicotine self-administration, extinction, and reinstatement. No sex differences for the age of initiation of behavior for nicotine self-administration, progressive ratio, extinction, and reinstatement. Mean ± SEM postnatal days across study conditions for male and female α6GG and α6CC. Circles represent females and squares represents males. [file Image_2.TIFF]
